# Rapidly Progressive Cutaneous Leishmaniasis in a Patient With HIV and Type 2 Diabetes Mellitus: A Case Report

**DOI:** 10.7759/cureus.90908

**Published:** 2025-08-24

**Authors:** Melanie Caballero Garcia, Juan M Rodriguez Marino, Carlos Valladares

**Affiliations:** 1 General Medicine, San Martín de Porres University, Lima, PER; 2 Internal Medicine, RWJBarnabas Health, Toms River, USA

**Keywords:** cutaneous leishmaniasis, hiv aids, immunocompromised patient, meglumine antimoniate, types 2 diabetes

## Abstract

Cutaneous leishmaniasis is a localized skin disease caused by protozoa of the genus *Leishmania*. We present the case of a 57-year-old man with controlled HIV infection and type 2 diabetes mellitus who presented with a rapidly progressing ulcerative lesion on the anterior part of the left leg. The lesion measured approximately 10 cm in diameter and 3 cm in depth and had been present for two months. The diagnosis was confirmed by a microscopic blood smear. The patient received intravenous meglumine antimoniate for 20 days without complications, achieving significant clinical improvement, considering the risk factors associated with coexisting diseases. Glycemic control improved during treatment, with no evidence of renal or hepatic toxicity. This case highlights the importance of early diagnosis and treatment of cutaneous leishmaniasis in immunosuppressed patients. Additionally, prompt identification and management of untreated comorbidities are essential to achieve a favorable outcome. Improving diagnostic sensitivity and specificity through advanced testing is crucial for early intervention and timely treatment initiation.

## Introduction

Cutaneous leishmaniasis (CL) is a neglected tropical disease caused by *Leishmania* species and transmitted by the bite of infected sandflies. In 2022, 205,990 cases of CL (205,653 autochthonous and 337 imported) were reported to the WHO, of which 99.98% of reported cases worldwide were treated in six high-incidence countries (Afghanistan, Brazil, Colombia, Morocco, Peru, and the Syrian Arab Republic) [1]. In Peru, CL is endemic in several regions, with the department of Madre de Dios among the most affected areas, particularly in rural and forested zones where vector exposure is high. Leishmaniasis is endemic in nearly 100 countries, and the total population at risk is estimated to be approximately 350 million [2]. Therefore, gaps in early diagnosis and timely treatment need to be urgently addressed.

CL in people with HIV varies based on their immune function. In individuals with well-controlled HIV and high CD4 counts, the presentation is similar to that of those without HIV, except for a higher relapse rate. In those with lower CD4 counts, dermal leishmaniasis can spread to the skin, mucous membranes, and viscera [3]. As with combined immunosuppression (HIV and type 2 diabetes mellitus (T2DM)), the approach is holistic and requires close monitoring to detect and respond promptly to any abnormal presentation or deviation from the usual clinical picture due to preexisting risk factors. All of these characteristics will be addressed in the clinical case.

## Case presentation

**Figure 1:**
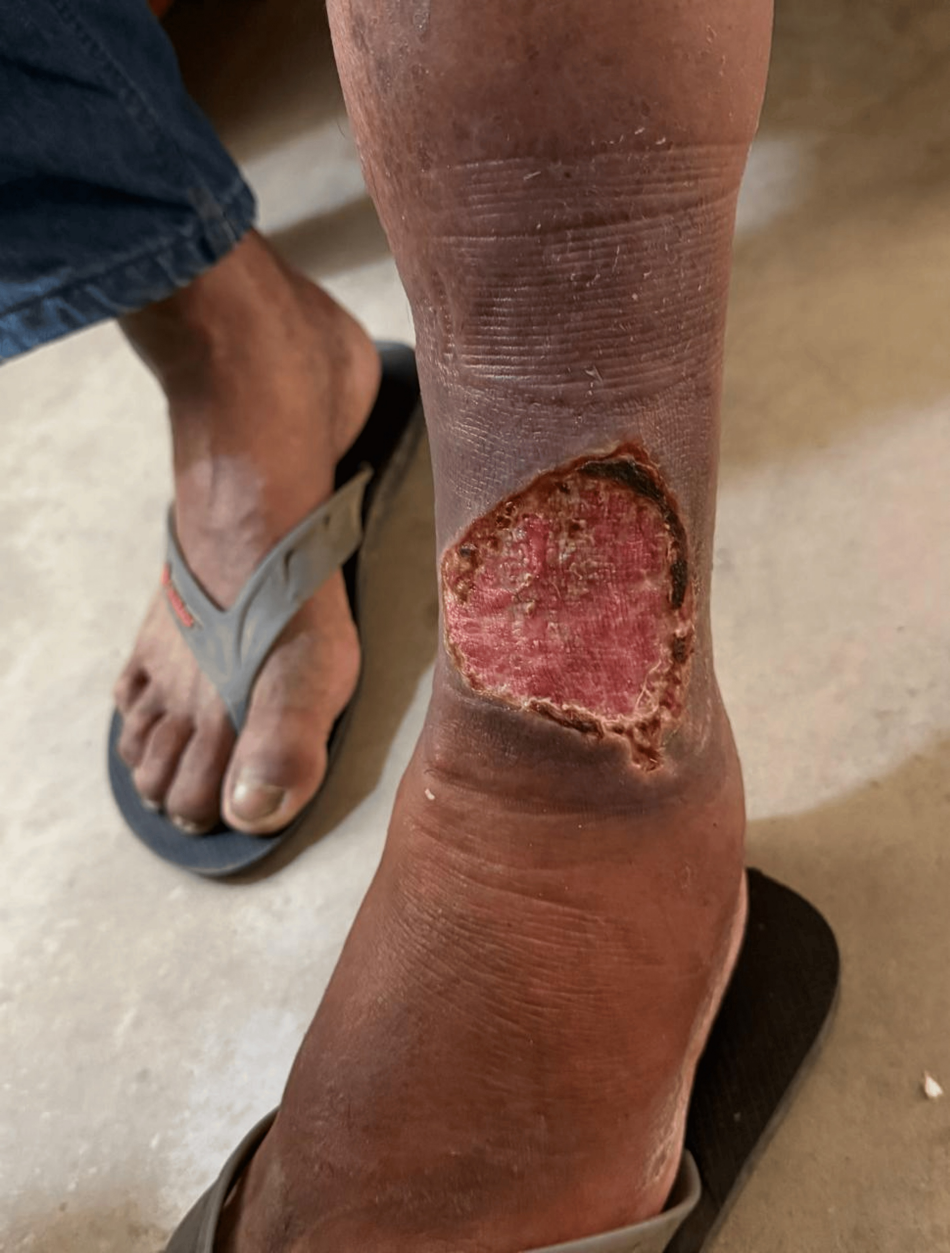
CL ulcer on the lower limb. The image shows a well-demarcated ulcer located on the medial aspect of the lower leg, just above the ankle. CL: cutaneous leishmaniasis.

In January 2025, a 57-year-old Peruvian mestizo man who works in the timber industry in the rural department of Madre de Dios, an endemic area for CL, likely acquired the infection during work-related activities in the virgin rainforest. He had a four-year history of HIV (on antiretroviral therapy, adherent) and five years of T2DM, as well as a recent diagnosis of hypertension. He presented to the outpatient clinic with an ulcerated lesion on his lower extremity. The patient reported that the lesion first appeared approximately two months ago as a small area no larger than 1 cm in diameter. Over time, it gradually enlarged, with a marked acceleration in growth during the past two weeks. Physical examination revealed a single, non-tender, circular ulcerated lesion on the lower two-thirds of the anterior aspect of the left calf, approximately 10 cm in diameter and 3 cm in depth, with raised and indurated edges, granulation tissue, erythema, and scant discharge (Figure 1).

**Figure 2:**
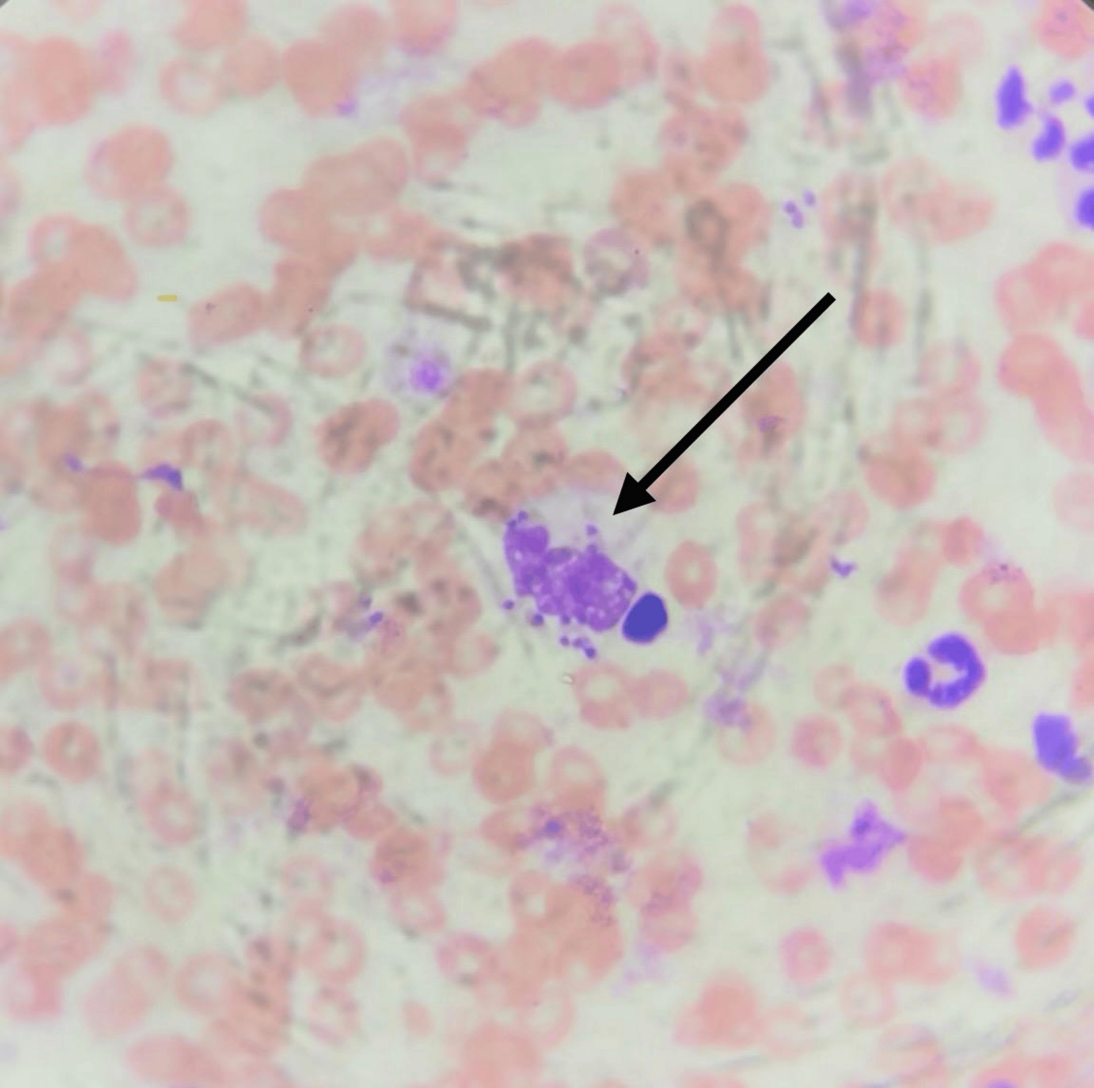
Microscopic identification of Leishmania amastigotes in a direct smear Direct smear of the cutaneous lesion stained with Giemsa, observed under 1000× magnification with oil immersion. The image shows macrophages containing multiple Leishmania amastigotes, identifiable by their oval shape, distinct nucleus, and rod-shaped kinetoplast.

Before initiating treatment, the patient underwent laboratory and diagnostic evaluations. Both the posteroanterior chest X-ray and electrocardiogram were unremarkable. Pre-treatment laboratory tests revealed a fasting blood glucose level of 186.6 mg/dL, normal liver function tests (AST, ALT, ALP, GGT), and normal renal function (creatinine and urea). To optimize glycemic control, the patient resumed treatment for T2DM with metformin 850 mg twice daily prior to obtaining a direct smear. The diagnosis of CL was ultimately confirmed through smear analysis of the lesion (Figure 2).

The patient initiated a 20-day course of intravenous meglumine antimoniate at a daily dose of 14.8 mL. By day 10 of treatment, laboratory results showed a fasting blood glucose of 148.7 mg/dL, creatinine of 1.2 mg/dL, and GGT of 68.7 U/L, with ALP, AST, and ALT remained within normal limits. The CD4 count was 257 cells/µL (Table 1). No evidence of renal or hepatic toxicity was observed. Clinically, the lesion showed progressive improvement throughout the course of treatment, with marked healing noted at the end of therapy.

**Table 1:** Laboratory findings. GGT: gamma-glutamyl transferase; ALP: alkaline phosphatase; AST: aspartate aminotransferase; ALT: alanine transaminase; CD4: clusters of differentiation 4.

Laboratory tests				
	Pre-treatment	During treatment	Post-treatment	Reference range
Hemoglobin	13.3 g/L	12.8 g/L	12.8 g/L	13.5–17.5 g/L
White blood count	6.2 x10³/µL	6.9 x10³/µL	7.1 x10³/µL	4.5–10.0 x10³/µL
Platelets	180 x10³/µL	225 x10³/µL	265 x10³/µL	150–400 x10³/µL
Lymphocytes	32%	24%	24%	25–35%
Glucose	186.6 mg/dL	148.7 mg/dL	160.1 mg/dL	60–100 mg/dL
GGT	45.8 U/L	68.7 U/L	45.5 U/L	11–61 U/L
ALP	109 UI/L	99 UI/L	110 UI/L	68–300 U/L
AST	15.7 U/L	31.6 U/L	20.7 U/L	10–38 U/L
ALT	19.1 U/L	39 U/L	20.8 U/L	7–41 U/L
Urea	46.4 mg/dL	40.6 mg/dL	46.4 mg/dL	10–50 mg/dL
Creatinine	0.95 mg/dL	1.2 mg/dL	1.3 mg/dL	0.6–1.1 mg/dL
CD4		257 cells/uL		500–1,500 cells/μL
HIV viral load	Undetectable	Undetectable		

The patient has been followed up for six months since the initiation of treatment. Progressive reduction in lesion size and improvement in surrounding inflammation have been observed, although complete re-epithelialization has not yet been achieved. The patient remains clinically stable, continues to adhere to antiretroviral therapy and metformin, and no new lesions have appeared during follow-up.

## Discussion

CL represents one of the most common clinical manifestations of *Leishmania* infection in endemic regions. Its clinical behavior can be significantly altered in patients with immunosuppression, such as those infected with the HIV and T2DM. The coexistence of these conditions poses a considerable diagnostic and therapeutic challenge due to the possibility of atypical clinical forms, poor therapeutic response, and higher relapse rates [4,5].

A more severe presentation of CL has been described in people with HIV, especially those with low CD4 lymphocyte counts, with a greater predisposition to dissemination to mucosal and visceral tissues. A count below 200 cells/μL is estimated to substantially increase the risk of visceral and refractory forms [6]. Although our patient had a CD4 count of 257 cells/μL, a value below the optimal threshold for immune protection, his undetectable viral load could have influenced the partial control of viral replication and, therefore, a more functional immune response, even within the context of relative immunosuppression [3].

It is also worth mentioning that patients with HIV have a higher likelihood of relapse, even after initial clinical resolution. Clinical guidelines recommend long-term follow-up in these cases, including periodic assessment of viral load, CD4 counts, and clinical examination of the affected area [7]. The low CD4/CD8 and CD4/CD3 ratios in this patient reinforce the need for close monitoring, as they are markers of persistent immunodysregulation, even when the viral load is undetectable [8].

On the other hand, T2DM has also been recognized as a factor that affects innate immunity, especially the phagocytic capacity of macrophages and the production of reactive oxygen species, fundamental mechanisms for controlling *Leishmania* infection [9]. This immunometabolic dysfunction could facilitate the rapid progression of skin lesions, as evidenced in this case. However, timely reinitiation of antidiabetic treatment before starting antileishmanial therapy may have contributed to better glycemic control, promoting a more effective therapeutic response.

Studies in experimental and human models have shown that patients with T2DM exhibit decreased macrophage and dendritic cell activity in response to parasitic infections, which could explain the greater clinical severity observed in these cases [10,11]. However, most research in this field has focused on visceral leishmaniasis, and there is still a significant gap in understanding the interaction between CL and chronic metabolic comorbidities such as diabetes [12].

Treatment with meglumine antimoniate, although historically used as first-line therapy in many endemic countries, poses risks of liver, kidney, and heart toxicity, especially in patients with comorbidities [13]. In this case, the patient tolerated treatment well for 20 days without evidence of hepatorenal toxicity, as indicated by creatinine and transaminase levels within normal ranges, with only a mild increase in GGT. This finding suggests that, with adequate monitoring, the conventional regimen can be used even in patients at increased risk.

The observed therapeutic success reinforces the need to individualize treatment for patients with complex comorbidities. Interdisciplinary evaluation, adjustment for comorbidities, and rigorous clinical follow-up are key to minimizing risks and optimizing treatment response [14]. Furthermore, this case highlights the importance of a comprehensive approach in which metabolic and virological control serve as pillars of management, beyond specific antiparasitic treatment.

The diagnosis of CL was confirmed by direct smear (direct microscopy), a rapid but limited sensitivity technique. Molecular methods such as PCR offer higher sensitivity and specificity, particularly in immunocompromised patients, and could complement or replace microscopy where resources permit [15,16]. Therefore, the implementation of PCR as part of the standard diagnostic process, whenever feasible, would enhance early diagnosis and improve patient outcomes, especially in settings where delayed diagnosis can lead to more severe disease progression.

In summary, this case reflects the inherent complexity of managing CL in patients with multiple immunosuppression. Despite factors often associated with a poor prognosis, such as low CD4 count, T2DM, and lesion size, the patient achieved significant clinical recovery with conventional treatment and appropriate follow-up, underscoring the importance of a comprehensive and personalized approach.

## Conclusions

CL can present aggressively in immunosuppressed patients (e.g., HIV and type 2 diabetes), regardless of whether comorbidities are controlled pharmacologically. Early diagnosis, close follow-up, and comprehensive management of underlying immunosuppressive conditions are essential for achieving a good prognosis.

The implementation of the PCR test as part of the standard diagnostic process due to its high sensitivity and specificity will be vital for timely diagnosis. While it is certainly more expensive than direct microscopy, its value in terms of early diagnosis can ensure better management and prevent complications, especially in immunocompromised patients who tend to present more aggressively. All of this is within the framework of the economic reach of different health systems worldwide.

## References

[1] (2025). Global report on neglected tropical diseases. https://www.who.int/publications/i/item/9789240091535.

[2] de Vries HJ, Schallig HD (2022). Cutaneous leishmaniasis: a 2022 updated narrative review into diagnosis and management developments. Am J Clin Dermatol.

[3] (2025). Guidelines for the Prevention and Treatment of Opportunistic Infections in Adults and Adolescents with HIV. https://www.ncbi.nlm.nih.gov/books/NBK586304/.

[4] Tarnas MC, Abbara A, Desai AN, Parker DM (2024). Ecological study measuring the association between conflict, environmental factors, and annual global cutaneous and mucocutaneous leishmaniasis incidence (2005-2022). PLoS Negl Trop Dis.

[5] Mann S, Frasca K, Scherrer S, Henao-Martínez AF, Newman S, Ramanan P, Suarez JA (2021). A review of leishmaniasis: current knowledge and future directions. Curr Trop Med Rep.

[6] van Griensven J, Diro E (2019). Visceral leishmaniasis: recent advances in diagnostics and treatment regimens. Infect Dis Clin North Am.

[7] Gómez-Zafra MJ, Navas A, Jojoa J, Murillo J, González C, Gómez MA (2025). Immune profile of the nasal mucosa in patients with cutaneous leishmaniasis. Infect Immun.

[8] Ron R, Martínez-Sanz J, Herrera S (2024). CD4/CD8 ratio and CD8+ T-cell count as prognostic markers for non-AIDS mortality in people living with HIV. A systematic review and meta-analysis. Front Immunol.

[9] Iwai N, Steib C, Marzo A, Lerret NM (2018). The role of hyperglycemia in CD4 T-cell survival and differentiation. Am Soc Clin Lab Sci.

[10] Mostafavi M, Sharifi I, Asadikaram G (2021). The impact of diabetes on cutaneous leishmaniasis: a case-control field assessment. Parasitol Res.

[11] Pazmiño FA (2025). Determination of the association between the presence of Leishmania virus 1 (LRV-1) in Leishmania spp. infecting parasites and the development of mucosal leishmaniasis in patients diagnosed with cutaneous leishmaniasis in Colombia. https://repositorio.unal.edu.co/handle/unal/78395.

[12] McIlwee BE, Weis SE, Hosler GA (2018). Incidence of endemic human cutaneous leishmaniasis in the United States. JAMA Dermatol.

[13] Madusanka RK, Silva H, Karunaweera ND (2022). Treatment of cutaneous leishmaniasis and insights into species-specific responses: a narrative review. Infect Dis Ther.

[14] (2025). Control of the leishmaniases: WHO TRS N°949. https://www.who.int/publications/i/item/WHO-TRS-949.

[15] Adams ER, Schoone G, Versteeg I (2018). Development and evaluation of a novel loop-mediated isothermal amplification assay for diagnosis of cutaneous and visceral leishmaniasis. J Clin Microbiol.

[16] Reimão JQ, Coser EM, Lee MR, Coelho AC (2020). Laboratory diagnosis of cutaneous and visceral leishmaniasis: current and future methods. Microorganisms.

